# High prevalence of risky sexual behaviour among key populations receiving antiretroviral therapy at a large HIV clinic in northern Uganda

**DOI:** 10.4314/ahs.v23i1.38

**Published:** 2023-03

**Authors:** Norbert Adrawa, Jonathan Izudi, Kenneth Nyeko, Emma Welikhe, Bennet Joseph Kizito, Francis Bajunirwe

**Affiliations:** 1 The AIDS Support Organization (TASO), Gulu Centre of Clinical Excellence, P. O. BOX 347, Gulu City; 2 Department of Community Health, Faculty of Medicine, Mbarara University of Science and Technology (MUST), P.O.BOX, 1410, Mbarara City

**Keywords:** Key populations, men who have sex with men, people who inject drugs, commercial sex workers, risky sexual behaviour

## Abstract

**Background:**

Risky sexual behaviour (RSB) among key populations pose a significant risk of human immunodeficiency virus (HIV) infection but remains understudied.

**Objectives:**

We assessed the prevalence and factors associated with RSB among key populations living with HIV (KPLHIV) in the post-conflict region of northern Uganda.

**Methods:**

We designed a cross-sectional study using secondary data, with the outcome as RSB defined as having multiple sexual partners, or condomless sex in the past 3 months, or sexual intercourse with a commercial sex worker in the past 3 months, or sexual intercourse under the influence of substance use in the past 3 months. We used modified Poisson regression to determine factors associated with RSB, reported as adjusted risk ratio (aRR) with 95% confidence interval (CI).

**Results:**

We studied 165 participants and 122 (73.9%) reported RSB and this was more likely among heterosexual females (aRR, 2.39; 95% CI, 1.54-3.71), the married (aRR, 1.92; 95% CI, 1.42-2.49) or separated participants (aRR, 1.47; 95% CI, 1.21-1.79), and transgender persons (aRR, 3.71; 95% CI, 2.05-6.71).

**Conclusions:**

RSB is highly prevalent among KPLHIV in northern Uganda so they should be targeted with HIV prevention and behavioural interventions to prevent potential HIV transmission to the general population.

## Introduction

Human Immunodeficiency Virus (HIV) infection rate among key populations is steadily increasing in sub-Saharan Africa^1^. Reports indicate that 25% of new HIV infections are among key populations, namely commercial sex workers, men who have sex with men, and people who use drugs including their sexual partners^1^. In Uganda, for example, about 85% of new HIV infections in 2018 were among commercial sex workers^1^,^2^. Sexual behaviours such as having multiple sexual partners, condomless sex, sex under the influence of alcohol, sex with commercial sex workers, and transactional sex increase the risk of HIV acquisition. Transactional sex is a non-marital, non-commercial sexual relationship largely motivated by the exchange of money, material support, or other benefits^3^. These factors, combined with social marginalization, criminalization, and a wide range of human rights abuses towards key population increase their vulnerability to HIV infection 4.

Between 1988 and 2009, northern Uganda suffered approximately two decades of civil unrest perpetrated by the rebels of the Lord's Resistance Army (LRA). Besides massive displacement, the insurgency led to most of the displaced persons engaging in commercial sex work as a survival strategy^5^,^6^. In the aftermath of the insurgency, the region experienced an enormous influx of commercial sex workers from the neighbouring regions and/or districts, due to economic boom^7^,^8^. Sexual mixing that involves high-risk sexual behaviour between key populations living with HIV (KPLHIV), and the general population is now a central public health problem in the region, because of its potential to drive and/or sustain HIV transmission in the general population. Many post-conflict regions such as Northern Uganda experience growth in key populations^5^, but there is limited research on their sexual behaviour. The identification of the determinants of risky sexual behaviour among this population is important, to inform the design of key public health HIV prevention strategies in Northern Uganda, and similar settings in sub-Saharan Africa.

Therefore, we conducted a study at a large, specialized, regional HIV clinic for key populations in Northern Uganda, to measure the frequency of risky sexual behaviour, and the associated factors among KPLHIV receiving antiretroviral therapy (ART).

## Materials

### Study setting, data source, and study population

This study was conducted at The AIDS Support Organization (TASO) Gulu HIV Clinic, which is located in Gulu City, 337 kilometres North of Uganda's Capital City, Kampala. TASO provides comprehensive HIV services to people living with HIV and more recently, it runs a large, specialized, regional HIV clinic for key populations in northern Uganda, which operates from Monday to Friday, 8.00 am to 5.30 pm. TASO is one of the largest and the first local organizations to respond to the HIV epidemic in Uganda and sub-Saharan Africa. TASO has 11 service Centers of Excellence spread across all regions in Uganda, with one state-of-the-art international training center known as TASO College of Health Sciences^9^. TASO has a robust and well-developed health information management system, allowing electronic capture of patient records and easy data retrieval. For this study, we used the existing web-based medical records database management system, the Key Population Tracker (KPT), to retrieve clinical and socio-demographic data on KPLHIV. The KPT was developed by the United States Agency for International Development (USAID), the main funder for key populations HIV programming at TASO Gulu. Our study population consisted of a census of KPLHIV, namely sex workers, men who have sex with men, people who use drugs, and transgender people registered in the KPT from inception on September 1, 2018 to August 30, 2020, and all of them were on ART.

### Study design and measurements

We used routinely collected patient data to design a cross-sectional study and reported the findings in accordance with the Strengthening of the Reporting of Observational studies in Epidemiology (STROBE) guideline^10^,^11^. The variables in the dataset used for the measurement of risky sexual behaviour included the following: 1) multiple sexual partners measured using data on the number of sexual partners in the last 6 months, categorized as <2 or ≥2; 2) condomless sex in the past 3 months determined using data on whether the participant had used a condom at all sexual encounters, categorized as yes or no; 3) sexual intercourse with a commercial sex worker in the past 3 months (yes or no), using data on whether the participant had reported having had sex with a commercial sex worker; 4) sexual intercourse under the influence of substance use in the past 3 months (yes or no), using data on history of sex under the influence of alcohol or substance abuse. Accordingly, risky sexual behaviour was defined as having multiple sexual partners, or condomless sex in the past 3 months, or sexual intercourse with a commercial sex worker in the past 3 months, or sexual intercourse under the influence of substance use, all measured as a dichotomous variable (no or yes).

The independent variables included the following: age measured in absolute years and later classified into two categories as ≤30 years and more than 30 years, sex at birth measured as male or female, and sexual orientation. Sexual orientation was defined as the feeling of emotional, romantic, or sexual attraction towards another person^12^. We categorized sexual orientation into the following 12,13: 1) Heterosexual males: romantic and physical attraction of males to females; 2) Heterosexual females: romantic and physical attraction of females to males; 3) Lesbian: romantic and physical attraction of females to females; 4) Gay: romantic and physical attraction of males to males; 5) Bisexual: the romantic and physical attraction to more than one sex; and 6)Transgender persons: people whose current sex identify differs from the sex at birth. We retrieved data on socio-demographics such as age, employment status, and level of education, and marital status, HIV discordant relationships, point of entry into HIV care as an outpatient department or outreach, internally displacement status, and drug use.

### Statistical analysis

The data analysis was performed in Stata version 15. In the univariate analysis, we computed frequencies and percentages for categorical variables such as sex, and means and standard deviations, or medians and interquartile ranges (IQR) for numerical data such as age. The outcome variable, risky sexual behaviour, was computed as the proportion of participants that reported having multiple sexual partners, or condomless sex in the past 3 months, or sexual intercourse with a commercial sex worker in the past 3 months, or sexual intercourse under the influence of substance use, with the study sample size as the denominator. In the bivariate analysis, we used the Chi-square test to assess differences in the proportion of risky sexual behaviour with categorical variables for larger cell counts (equal or greater than five) or the Fisher's exact test when the cell count was smaller (less than five). We used the student's t-test to assess mean differences in numerical variables like age when we compared the subgroups.

We used a modified Poisson regression analysis with robust error variance because of the potential for over-dispersion in the outcome variable and since the odds ratio would overestimate the degree of association 14, 15. We reported both unadjusted and adjusted risk ratio (RR) with the corresponding 95% confidence interval (CI). Variables with probability values less than 5% at the bivariate analysis were considered for multivariable regression analysis. We modelled risky sexual behaviour as a function of age, sex, level of education, marital status, sex orientation, and category of KPLHIV. We excluded collinear variables as determined by a variance inflation factor ≥10. We also excluded variables that did not improve the model fit as measured by the log-likelihood to establish a parsimonious model. We performed a goodness-of-fit test for the final model and determined if both the deviance statistic and the Pearson statistic confirm good model fit.

### Ethical issues

We received ethical review and approval from TASO Research Ethics Committee (TASO-REC) and the approval number is TASOREC/045/2020-UG-REC-009. We also received a waiver of informed consent to retrieve the secondary data. To maintain participant anonymity, we had no access to personal identifiers like names and physical addresses. We were only granted access to anonymous patient records/dataset.

## Results

### General characteristics of participants

We retrieved records for 165 participants and Table 1 summarizes the general characteristics of the participants. The mean age was 29.1 years (SD = 5.4) and 95 (57.6%) were aged 30 years or younger, 84 (50.9 %) were females, 99 (60.0%) had no formal education or had ended at primary school, and 74 (44.8%) were single or never married. Two thirds or 110 (66.7%) had rolled to the HIV program through the outpatient department, 5 (3.3%) were internally displaced persons, and 32 (19.4%) were in HIV discordant relationships. Overall, the categories of KPLHIV were distributed as follows: 107 (64.8%) sex workers, 20 (12.1%) men who have sex with men, 28 (17.0%) people who use drugs, and 10 (6.1 %) transgender people.

**Table 1:** Characteristics of participants

		Overall (n=165)
Characteristics	Level	n (%)
Age group in years	≤30	95 (57.6)
	>30	70 (42.4)
	mean (SD)	29.1 (5.4)
Sex of participant at birth	Female	84 (50.9)
	Male	81 (49.1)
Level of education	None/primary school	99 (60.0)
	Secondary school	34 (20.6)
	Tertiary	32 (19.4)
Marital status	Single	74 (44.8)
	Married	33 (20.0)
	Separated	58 (35.2)
Type of job	None/refused to report	69 (41.8)
	Long distance truck driver/seasonal worker	77 (46.7)
	Other jobs	19 (11.5)
Point of entry into HIV care	Outpatient department	110 (66.7)
	Outreach	55 (33.3)
Internally displaced person	No	146 (96.7)
	Yes	5 (3.3)
In HIV discordant relationship	No	133 (80.6)
	Yes	32 (19.4)
Categories of KPLHIV	Commercial sex worker	107 (64.8)
	Men who have sex with men	20 (12.1)
	People who use drugs	28 (17.0)
	Transgender	10 (6.1)

### Risky sexual behaviour among the study participants

Our data show that 103 (62.4%) participants had sexual intercourse with a commercial sex worker in the past 3 months, 14 (8.5%) had multiple sexual partners, 151 (91.5%) had condomless sex in the past 3 months, and 121 (73.3%) had sexual intercourse under the influence of substance use in the past 3 months. Overall, 122 (73.9%) reported at least one risky sexual behaviour (Table 2).

**Table 2:** Risky sexual behaviour among the study participants

Characteristics	Frequency	n (%)
Sexual intercourse with a commercial sex worker in the past 3 months	No	62 (37.6)
	Yes	103 (62.4)
Has multiple sexual partners	No	151 (91.5)
	Yes	14 (8.5)
Condomless sex in the past 3 months	No	14 (8.5)
	Yes	151 (91.5)
Sexual intercourse under the influence of substance use	No	44 (26.7)
	Yes	121 (73.3)
Engaged in risky sexual behaviour	No	43(26.1)
	Yes	122 (73.9)
**Overall**		**165 (100.0)**

### Bivariate analysis of differences between risky sexual behaviour and participant characteristics

Table 3 shows that the following categories of participants had the highest proportion of risky sexual behaviour: age category less or equals 30 years (62.3%), females (61.5%), heterosexual females (73.0%), no formal education or some primary school education (65.6%), separated marital relationship (43.4%), enrolled through the outpatient department (67.2%), none internally displaced persons (95.4%), and seasonal workers (43.4%). On average, participants with risky sexual behaviour were younger compared to those without risky sexual behaviour: 28.6±5.7 versus 30.6±4.1, p = 0.034, respectively. We observed statistically significant differences in risky sexual behaviour concerning age categories (*p* = 0.039), sex at birth (p<0.001), sexual orientation (*p*<0.001), categories of KPLHIV (p<0.001), level of education (*p* = 0.020), and marital status (p<0.001). Regarding the categories of KPLHIV, those engaged in risky sexual behaviour were mainly commercial sex workers (76.2%), followed by transgender persons (9.7%), then men who have sex with men (8.2%), and lastly people who use drugs (7.4%).

**Table 3:** Bivariate analysis of differences in risky sexual behaviour with participant characteristics

			Risky sexual behaviour		
		Overall (n= 165)	No (n = 43)	Yes (n = 122)	
Characteristics	Level	(n, %)	(n, %)	(n, %)	p value
Age group (years)	-30 verses >30	95 (57.6)	19 (44.2)	76 (62.3)	0.039
		70 (42.4)	24 (55.8)	46 (37.7)	
	mean (SD)	29.1 (5.4)	30.6 (4.1)	28.6 (5.7)	0.034
Sex at birth	Female	84 (50.9)	9 (20.9)	75 (61.5)	<0.001
	Male	81 (49.1)	34 (79.1)	47 (38.5)	
Sexual orientation	Bisexual	15 (9.1)	5 (11.6)	10 (8.2)	<0.001
	Gay	15 (9.1)	10 (23.3)	5 (4.1)	
	Heterosexual females	94 (57.0)	5 (11.6)	89 (73.0)	
	Heterosexual males	41 (24.8)	23 (53.5)	18 (14.8)	
Categories of KPLHIV	People who use drugs	28 (17.0)	19 (44.2)	9 (7.4)	<0.001
	Men who have sex with men	20 (12.1)	10 (23.3)	10 (8.2)	
	Commercial sex worker	107 (64.8)	14 (32.6)	93 (76.2)	
	Transgender	10 (6.1)	0 (0.0)	10 (9.7)	
Level of education	None/primary	99 (60.0)	19 (44.2)	80 (65.6)	0.020
	Secondary	34 (20.6)	10 (23.3)	24 (19.7)	
	Tertiary	32 (19.4)	14 (32.6)	18 (14.8)	
Type of job	None/refused to report	69 (41.8)	14 (32.6)	55 (45.1)	0.327
	Seasonal worker	77 (46.7)	24 (55.8)	53 (43.4)	
	Other jobs	19 (11.5)	5 (11.6)	14 (11.5)	
Marital status	Single	74 (44.8)	33 (76.7)	41 (33.6)	<0.001
	Married	33 (20.0)	5 (11.6)	28 (23.0)	
	Separated	58 (35.2)	5 (11.6)	53 (43.4)	
In HIV discordant relationship	No	133 (80.6)	38 (88.4)	95 (77.9)	0.134
	Yes	32 (19.4)	5 (11.6)	27 (22.1)	
Point of entry into HIV care	Outpatient department	110 (66.7)	28 (65.1)	82 (67.2)	0.950
	Outreach	55 (33.3)	15 (34.9)	40 (32.8)	
Internally displaced person	No	146 (96.7)	43 (100.0)	103 (95.4)	0.352
	Yes	5 (3.3)	0 (0.0)	5 (4.6)	

### Factors associated with risky sexual behaviour in the unadjusted and adjusted analyses

In the unadjusted analysis (Table 4), risky sexual behaviour was significantly lower among males compared to females (RR, 0.65; 95% CI, 0.53-0.79) and among those who had attained tertiary level of education compared to those without formal education or those with some primary school education (RR, 0.70; 95% CI, 0.50-0.96). Conversely, risky sexual behaviour was significantly higher among participants who had married (RR, 1.53; 95% CI, 1.19-1.97) or separated (RR, 1.65; 95% CI, 1.32-2.05) compared to the single or never married, and among commercial sex workers (RR, 2.70; 95% CI, 1.57-4.66) and transgender persons (RR, 3.11; 95% CI, 1.81-5.34) compared to the people who use drugs. In the adjusted analysis (Table 4), the level of education did not improve the model fit while sex was collinear so they were excluded to achieve a final parsimonious model with deviance goodness-of-fit value of 48.94 and person goodness-of-fit value of 38.93, both with degrees of freedom of 155 and statistically insignificant p-value.

**Table 4:** Factors associated with risky sexual behaviour at unadjusted and adjusted analyses

		Unadjusted analysis		Adjusted analysis	
Characteristics	Level	RR	95% CI	aRR	95% CI
Age group in years	=30	1		1	
	>30	0.82	(0.67,1.00)	1.04	(0.87,1.24)
Sex at birth	Female	1			
	Male	0.65***	(0.53,0.79)		
Sexual orientation	Bisexual	1		1	
	Gay	0.50	(0.22,1.12)	0.75	(0.39,1.44)
	Heterosexual female	1.42	(0.99,2.04)	2.39***	(1.54,3.71)
	Heterosexual male	0.66	(0.40,1.08)	1.44	(0.81,2.56)
Educational level	None/primary	1			
	Secondary	0.87	(0.69,1.11)		
	Tertiary	0.70*	(0.50,0.96)		
Marital status	Single	1		1	
	Married	1.53***	(1.19,1.97)	1.92***	(1.47,2.49)
	Separated	1.65***	(1.32,2.05)	1.47***	(1.21,1.79)
Categories of KPLHIV	People who use drugs	1		1	
	Men who have sex with men	1.56	(0.78,3.12)	1.98	(0.84,4.64)
	Commercial sex worker	2.70***	(1.57,4.66)	1.67	(0.89,3.14)
	Transgender	3.11***	(1.81,5.34)	2.93**	(1.49,5.74)

**Note:** 1) Risk ratios are exponentiated coefficients at 5% significance level with the 95% confidence intervals in brackets; 2) RR: Unadjusted risk ratio; 3) ARR: Adjusted risk ratio; 5) * *p* < 0.05, ** *p* < 0.01, *** *p* < 0.001; 4)

Our final model showed that risky sexual behaviour was more likely among heterosexual females (Adjusted Risk Ratio (aRR), 2.39; 95% CI, 1.54-371) compared to bisexuals, married (aRR, 1.92; 95% CI, 1.42-2.49) or separated (aRR, 1.47; 95% CI, 1.21-1.79) compared to those single/never married participants, and transgender persons (aRR, 3.71; 95% CI, 2.05-6.71) compared to people who use drugs.

## Discussion

We studied the frequency and determinants of risky sexual behaviour among KPLHIV in northern Uganda. Our data show that risky sexual behaviour is more frequent in this post-conflict region. We found that risky sexual behaviour is more likely among heterosexual females, the married or separated participants, including transgender persons. The higher frequency of risky sexual behaviours is consistent with data from other key populations in sub-Saharan Africa.

For instance, findings from studies on risky sexual behaviour among men who have sex with men in Rwanda^16^, ^17^ and among Kenyan women who inject drugs 18 also show risky sexual behaviour is more prevalent. Another study conducted among men who have sex with men in Uganda reports a high prevalence of condomless anal sex, commercial sex work, and a tendency to have multiple steady or casual partners 19, which is consistent with our findings.

The finding that risky sexual behaviour is more likely among heterosexual females compared to the bisexuals is in agreement with the findings of previous studies that report males are less likely to use condoms compared to females 20, 21. Socio-cultural differences between males and females, with females being more submissive to the sexual demands of males also explain the findings. Another supporting evidence comes from a previous Ugandan study 22 which reports the social environment as an independent risk factor for HIV vulnerability compared to individual-level factors. Our findings underscore a need for providing sufficient health information on the risk of acquisition of HIV resistant strains and other sexually transmitted infections (STI) by engaging in risky sexual behaviours.

Our study shows an increased likelihood of risky sexual behaviour among married or separated participants compared to the single or never married participants. This finding is consistent with an earlier study in Ethiopia that linked risky sexual behaviour such as condomless sexual intercourse to the desire to conceive and bear children among HIV infected couples 23. Nonetheless, we did not collect data on fertility desires from the participants. More research is needed to explain this finding within our context.

Our finding that risky sexual behaviour is more likely among transgender persons is consistent with earlier evidence that HIV infection in Uganda is driven by key populations 1, 2. Evidence about transgender persons and risky sexual behaviour within the Ugandan context is scarce. However, previous study in Nepal reports that transgender women are more likely to practice condomless vaginal and anal sex, including condomless sex with multiple partners 24. Elsewhere, another study reports that transgender adolescent females are more likely to practice inconsistent condom use following HIV diagnosis 25. Overall, male and female transgender persons are more likely to have multiple sexual partners 26, both within their networks and in the general population and this increases the risk of HIV transmission. This finding emphasizes a need for targeted HIV prevention strategies.

### Implications of findings for KPLHIV and HIV programming

The high prevalence of risky sexual behaviour among KPLHIV in this study has several implications. Given that older men who have sex with men do occasionally engage in sexual relations with women to minimize suspicion and social stigma and that younger men who have sex with men also engage in transactional sex 16, these practices may drive HIV transmission in the general population. A recent study in South Africa reports that transactional sex remarkably increases the risk of HIV acquisition among young women and that the risk of HIV acquisition is higher when there is frequent exchange of money and/or gifts 27. Adherence to ART is necessary to achieve virologic suppression. Data on adherence challenges among men who have sex with men (MSM) and lesbians, gay, bisexual, transgender, and intersex (MSM and LGBTI), as key populations in Uganda show that they face psychological and physical challenges due to daily swallowing of HIV medications and strict adherence^28^.

To mitigate HIV transmission in the general population by KPLHIV, there is a need to strengthen adherence to antiretroviral therapy (ART) since people with undetectable viral load cannot transmit the virus to another person through sexual intercourse^29^,^30^.

Poor adherence may result in the emergence of drug-resistant strains of HIV 31 which may also be transmitted^32^. One large study conducted among rural and urban cohorts of PLHIV in Uganda showed that longer ART duration is associated with a lower likelihood of risky sexual behaviour^33^. A South African study reports that consistent condom use and condom use at last sexual intercourse is more likely among people on ART compared to the ART naïve^34^. However, these observations were in the general population of PLHIV. Another study conducted in the United States reports that HIV-infected heterosexual men who have difficulties in adhering to ART are more likely to practice risky sexual behaviour and thus might benefit from counselling about risky sexual behaviours^35^. Whereas there is strong evidence of a relationship between ART and risky sexual behaviour among PLHIV, this information remains scarce among KPLHIV in sub-Saharan Africa. Therefore, there is a need to understand the association between ART and risky sexual behaviours. For example, there is a need to know whether ART duration (ART experienced versus ART inexperienced) is associated with risky sexual behaviours or a change in risky sexual behaviours. In addition, more evidence is needed to examine concerns on whether viral load suppression status (suppressed versus unsuppressed) influences risky sexual behaviour or changes in risky sexual behaviour among KPLHIV. This evidence is well known in the general population of PLHIV but remains understudied among KPLHIV. Our study did not explore these associations due to data limitations so we recommend that this topic should be the focus for prospective studies in this setting and elsewhere. At the program level, there is a need to identify approaches for screening for risky sexual behaviour and developing targeted interventions including integrating such interventions into HIV clinical care for KPLHIV.

### Study strengths

Our study has some important strengths. It is one of the first studies among the key populations in the post-conflict region of northern Uganda. The study was conducted at the only, larger, specialized HIV clinic for key populations in northern Uganda. The findings therefore provide a good picture of risky sexual behaviour for many similar settings in sub-Saharan and beyond. Our sample size is reasonable given the difficulty in reaching KPLHIV due to stigma and discrimination in the community, the existence of punitive laws and legislations, and the tendency for KPLHIV not to use existing health services 4.

## Study limitations

Our study has some limitations. We analysed secondary data so we had no information on the duration and level of adherence to ART yet it is associated with risky sexual behaviour (36). Our study population consisted of predominantly semi-urban and rural participants, so the likelihood that the findings are generalizable to KPLHIV in an urban setting might be limited due to differences in economic and behavioural characteristics. There is also the possibility of social desirability bias, and data recording and transcription errors that could have influenced the precision of the estimates.

## Conclusions and recommendations

Our study shows that a high proportion of KPLHIV engage in risky sexual behaviour, posing an increased risk for HIV transmission in the general population. Risky sexual behaviour is more likely among heterosexual females compared to bisexual, married or separated KPLHIV compared to the single/never married, and transgender persons compared to people who use drugs. We recommend that KPLHIV should be provided with targeted HIV prevention messages to enhance safer sexual practices.
